# Supplementary material for: Splice-Junction-Based Mapping of Alternative Isoforms in the Human Proteome
Source: Cell Rep. Author manuscript; Available in PMC 2020 Jan 15. (PMC6961840; doi:10.1016/j.celrep.2019.11.026)
Supplement: 2 [file NIHMS1546469-supplement-2.zip › DF1/PXD009737/TestisTrypsin/TestisTrypsin_68_ARHGAP26_RTGGSGWKPWMAGNLCAVGQHWLQH.pdf]

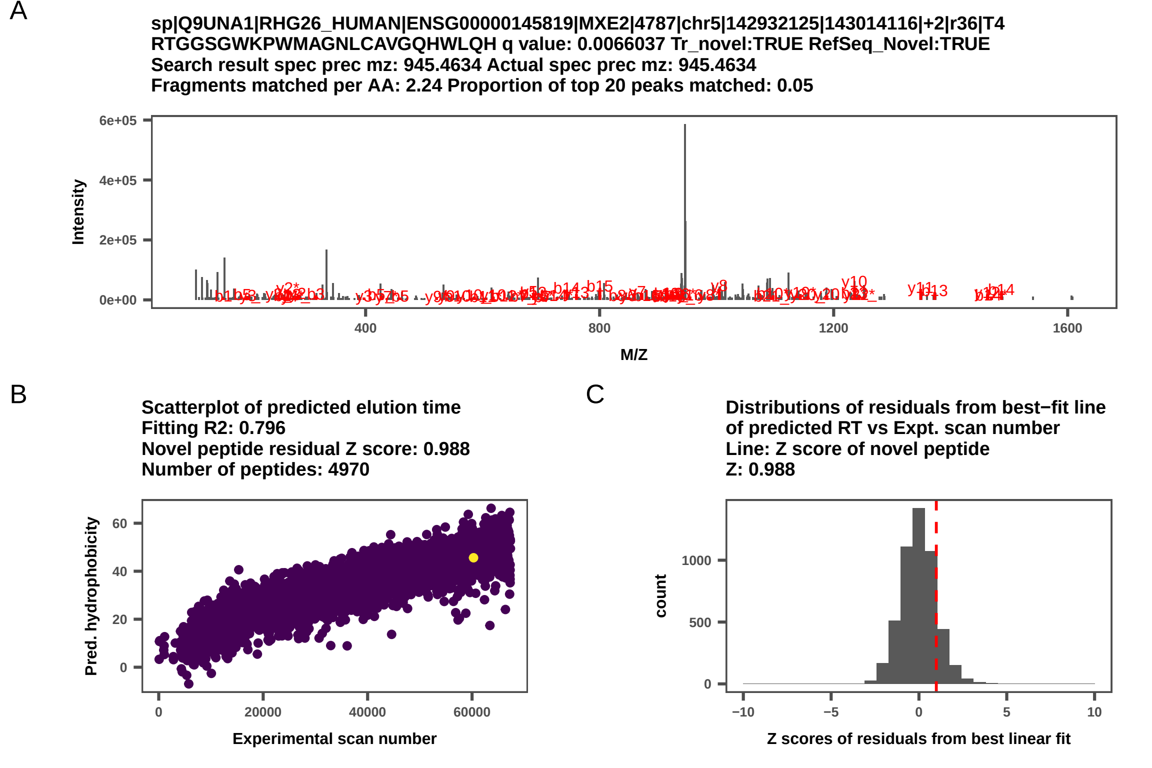
A
sp|Q9UNA1|RHG26_HUMAN|ENSG00000145819|MXE2|4787|chr5|142932125|143014116|+2|r36|T4
RTGGSGWKPWMAGNLCAVGQHWLQH q value: 0.0066037 Tr_novel:TRUE RefSeq_Novel:TRUE
Search result spec prec mz: 945.4634 Actual spec prec mz: 945.4634
Fragments matched per AA: 2.24 Proportion of top 20 peaks matched: 0.05
Intensity
6e+05
4e+05
2e+05
0e+00
400
800
1200
1600
M/Z
B
Scatterplot of predicted elution time
Fitting R2: 0.796
Novel peptide residual Z score: 0.988
Number of peptides: 4970
Pred. hydrophobicity
60
40
20
0
0
20000
40000
60000
Experimental scan number
C
Distributions of residuals from best−fit line
of predicted RT vs Expt. scan number
Line: Z score of novel peptide
Z: 0.988
count
1000
500
0
−10
−5
0
5
10
Z scores of residuals from best linear fit
